# An abrupt regime shift of bacterioplankton community from weak to strong thermal pollution in a subtropical bay

**DOI:** 10.3389/fmicb.2024.1395583

**Published:** 2024-04-30

**Authors:** Zhiyi Shan, Haiming Chen, Yuan Deng, Dan He, Lijuan Ren

**Affiliations:** ^1^Department of Ecology and Institute of Hydrobiology, Jinan University, Guangzhou, China; ^2^Center for Evolution and Conservation Biology, Southern Marine Science and Engineering Guangdong Laboratory (Guangzhou), Guangzhou, China

**Keywords:** bacterioplankton diversity, thermal pollution, regime shift, a subtropical bay, community assembly

## Abstract

Thermal pollution from the cooling system of the nuclear power plants greatly changes the environmental and the ecological conditions of the receiving marine water body, but we know little about their impact on the steady-state transition of marine bacterioplankton communities. In this study, we used high-throughput sequencing based on the 16S rRNA gene to investigate the impact of the thermal pollution on the bacterioplankton communities in a subtropical bay (the Daya Bay). We observed that thermal pollution from the cooling system of the nuclear power plant caused a pronounced thermal gradient ranging from 19.6°C to 24.12°C over the whole Daya Bay. A temperature difference of 4.5°C between the northern and southern parts of the bay led to a regime shift in the bacterioplankton community structure. In the three typical scenarios of regime shifts, the steady-state transition of bacterioplankton community structure in response to temperature increasing was more likely consistent with an abrupt regime shift rather than a smooth regime or a discontinuous regime model. Water temperature was a decisive factor on the regime shift of bacterioplankton community structure. High temperature significantly decreased bacterioplankton diversity and shifted its community compositions. *Cyanobium* and *Synechococcus* of Cyanobacteria, NS5 marine group of Bacteroidota, and *Vibrio* of Gammaproteobacteria were found that favored high temperature environments. Furthermore, the increased water temperature significantly altered the community assembly of bacterioplankton in Daya Bay, with a substantial decrease in the proportion of drift and others, and a marked increase in the proportion of homogeneous selection. In summary, we proposed that seawater temperature increasing induced by the thermal pollution resulted in an abrupt regime shift of bacterioplankton community in winter subtropical bay. Our research might broad our understanding of marine microbial ecology under future conditions of global warming.

## 1 Introduction

Regime shifts are present in most natural ecosystems and complex social systems (Rial et al., 2004; Schröder et al., 2005; Brock and Carpenter, 2006; Dakos et al., 2019; Hughes et al., 2019). Regime shifts in ecosystems could be regarded as a sudden change in ecosystem status where core ecosystem structures, functions and processes are fundamentally shifted at an environmental threshold (Lees et al., 2006; Dakos et al., 2019; Berdugo et al., 2020). These regime shifts are generally driven by external perturbations or the system’s internal dynamics following the environmental changes (Scheffer et al., 2001; Guttal and Jayaprakash, 2007; Lu and Hedin, 2019). The external perturbations include global change such as climate warming, overexploitation, invasive species, and eutrophication in both freshwater and marine ecosystems (Hare and Mantua, 2000; Schröder et al., 2005; Andersen et al., 2009; McMahon et al., 2015; Rocha et al., 2018). In most cases, these external perturbations can suddenly tip ecosystems to an alternative stable state, and according to this, they may be early warned by warning signal of the regime shifts (Guttal and Jayaprakash, 2008; McGlathery et al., 2013; Wernberg et al., 2016; Putten et al., 2019). The existence of an abrupt change-point in ecological system is a necessary condition for warning a regime shift. There are three typical scenarios for regime shifts: smooth, abrupt, and discontinuous. Two of them, smooth and abrupt, can be reversibly in response to the change in environment, however the other one was not reversible (Andersen et al., 2009; Dakos et al., 2019). The first one is smooth pressure-status relationships (smooth regime shift). In this type of regime shifts, environmental conditions often change gradually, even linearly. Ecosystem in this state tend to change in a smooth way. The second one is threshold-like state responses (abrupt regime shift), in which the ecosystem state shifts to another state suddenly after the environmental condition exceeds a threshold. The last one is bistable systems with hysteresis (discontinuous regime shift), which the hysteresis loop linking the ecosystem state to the environmental driver results in jumps between two alternative states when the driver is first slowly increased and then decreased again (DeYoung et al., 2004; Lees et al., 2006; Su et al., 2019; Martin et al., 2020; Moi et al., 2021).

Most studies of steady-state transition are focused on marine fish (Rahman et al., 2019; Perälä et al., 2020), phytoplankton, zooplankton (Djeghri et al., 2023), sediment (Dijkstra et al., 2019), or ecosystem (Szalaj et al., 2021), but fewer research investigates bacterioplankton community. Bacterioplankton is an indispensable part of the plankton food web and plays a crucial role in the biogeochemistry cycle by regenerating nutrients and decomposing organic matter (Ruiz-González et al., 2015). The temperature optimum varies among different bacterioplankton populations, and thus water temperature is one of the main driving factors that determine the community structure of bacterioplankton (Daufresne et al., 2009; Adams et al., 2010; Ren et al., 2013, 2017). A small increase in water temperature will indirectly lead to changes in the abundance and biomass of bacterioplankton (Christoffersen et al., 2006; Özen et al., 2013). Simultaneously, environmental warming will enhance the complexity of species associations, leading to changes in the diversity, structure and function of bacterial community (Zhu et al., 2019; Zhao et al., 2020; Yuan et al., 2021). In the study of Xingyun Lake, it had been shown that water temperature was a major factor affecting plankton biomass and community succession, and meanwhile, temperature was a major force in the regime shift (Wang et al., 2010). Rosero-López and his colleague found that the increase in temperature significantly enhanced the abundance of cyanobacteria and promoted the transition of regime shifts in aquatic ecosystems (Rosero-López et al., 2022). Temperature was thus a significant environmental factor affecting the diversity, community structure and function of bacterioplankton, and it might be also a factor that promoted regime shifts of bacterioplankton community in water bodies. There may be an early warning temperature that serves as an indicator for steady state transformation (Zhang et al., 2015; Anderson et al., 2018; Rosero-López et al., 2022).

Daya Bay is a typical subtropical bay locating in the north of the South China Sea (Tang et al., 2018). Long-term thermal pollution from the cooling systems of the nuclear power plants generates a temperature gradient in the Daya Bay, making it an ideal experimental area for studying regime shift driven by seawater temperature increasing. Long-term thermal pollution can cause changes in the bacterioplankton community compositions (Niu et al., 2011; Eiler et al., 2012; Ren et al., 2019), and the increase in water temperature as an external driving force might promote the steady-state transformation of bacterial community structure. Therefore, in this study, we aimed to answer (i) whether there is a steady-state transition driven by the increase in seawater temperature caused by the long-term thermal pollution from the nuclear power plants? (ii) which of the three typical models (smooth regime shift, abrupt regime shift, and discontinuous regime shift) does the steady-state transformation of bacterial community structure match when in response to an external driving force of seawater temperature increasing? (iii) how do the diversity and structure of bacterioplankton communities change after steady-state transformation. Our research might contribute to a better understanding of marine microbial ecology under future conditions of global warming.

## 2 Materials and methods

### 2.1 Study area

Daya Bay is located in the northern South China Sea (22°30′N to 22°50′N, 114°29′E to 114°49′E) between Shenzhen and Huizhou on the south of Guangdong Province, China. The depth in the middle of the Daya bay is about 10 meters while the depth of the bay mouth is match up to 20 meters. The Daya Bay belongs to subtropical monsoon climate, with an average annual air temperature of 22°C. Our water samples were collected on December 1st (2021) when the air temperature was from 9°C to 18°C. The northeast and west of Daya Bay were mostly used for aquaculture, the north was petrochemical area, the east was tourism development zone, and the southwest was located by the Daya Bay nuclear power station which began operation with an installed capacity of 1.968 million kilowatts in 1994 and with an installed capacity of 2.172 million kilowatts in 2010 (data provided by South China Nuclear and Radiation Safety Supervision Station of the Ministry of Ecology and Environment). Thermal effluents from the two nuclear power plants cooling systems have been present in the Daya Bay for more than 20 years and have generated a comparatively stable temperature gradient (a temperature increase from 0 to 5°C at the sampling time).

### 2.2 Water sample collection and environmental determination

**Figure 1:**
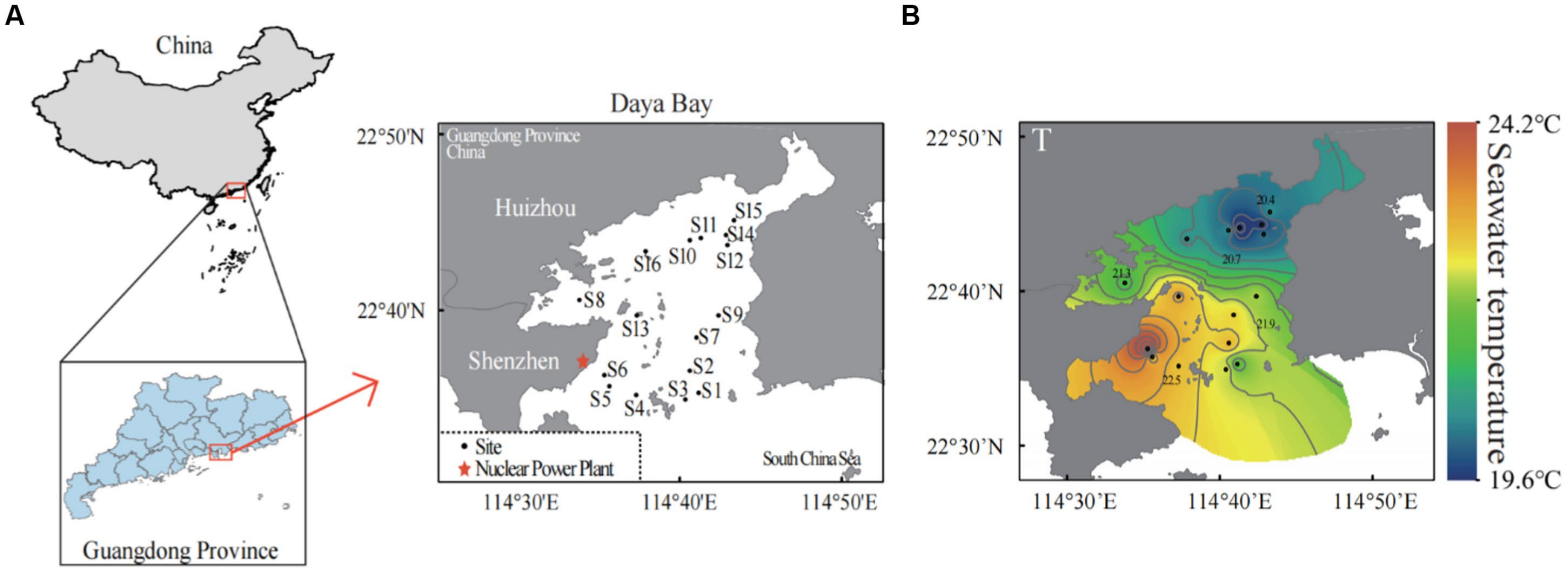
Sampling sites in the subtropical Daya Bay on the northern coast of the South China Sea. A total of 16 sites were sampled in the bay area: seven scattered sites in the north (S8, S10–S12, S14–S16), two scattered sites in the middle of the bay area (S9, S13), seven scattered sites in the south (S1–S7), and site S6 is the site near the outlet of thermal effluents from nuclear power plants **(A)**. The distributions of seawater temperature in the Daya Bay **(B)**.

We totally set 16 sampling sites according to the functional area of Daya Bay. Seven sites were located in the south of the bay which were more affected by the thermal effluents from the nuclear power plants (S1–S7), and S6 was sampling site that was closest to the nuclear power plants. There were two sites in the middle of the bay (S9 and S13) and the remaining 7 sites were in the north of the bay (S8, S10–S12, S14–S16) (Figure 1A). We collected three replicates at each sampling site. The sea surface temperature, salinity, dissolved oxygen and pH were measured in each replicate site by using multi-parameter water quality analyzer (YSI 6600, Yellow Spring, OH, United States; Xu et al., 2010). In each replicate site, 5-liter surface seawater was collected by using 0.2-μm-pore-size Isopore filters (Millipore, Billerica, MA, United States). The filters were unsealed in a clean bench and stored at −80°C in refrigerator until further analyses. Another 500 mL of surface seawater in each replicate site was collected for the nutrient measurements including ammonium nitrogen (NH_4_^+^), nitrate nitrogen (NO_3_^−^), nitrite nitrogen (NO_2_^−^), silicate (SiO_3_^2−^) and soluble reactive phosphorus (SRP). All these nutrients were determined using a UV–visible spectrophotometer (UV2450; Shimadzu, Tokyo, Japan) according to marine monitoring specifications (General Administration of Quality Supervision, 2007).

### 2.3 DNA extraction and PCR amplification, high-throughput sequencing and data processing

We use PowerWater DNA isolation Kit (MoBio Laboratories, CarIsbad, CA) to extract the Genomic DNA from the biomass collected on the filters, then purify the Genomic DNA by using PowerClean DNA Clean-up Kit (MoBio Laboratories, CarIsbad, CA, United States). Using a NanoDrop 2000 spectrophotometer (Thermo Scientific, Wilmington, DE Ren et al., 2019) to quantify the DNA and determine its quality. With 60 ng DNA as template, PCR amplification was performed in the V3 and V4 hypervariable regions of bacterial 16S rRNA genes, and the amplification primers were F515(5′-GTGCCAGCMGCCGCGGTAA-3′) and R907(3′-CCGTCAATTCCT TTGAGTTT-5′). We added a unique 12-mer tag to the 5′ end of each single DNA sample for pooling multiple samples in one Illumina sequencing run. The cycling conditions of PCR reactions included 94°C for 5 min initially, soon afterwards 30 cycles of denaturation for 30 s at 94°C, 30 s at 52°C for annealing, 30 s at 72°C for extension, and 10 min at 72°C for a final extension. The PCR products were visualized on 1% agarose gels by agarose gel electrophoresis, and then used the PicoGreen dsDNA assay kit (Invitrogen Corporation, CarIsbad, CA, United States) to quantify the positive amplicons. Zymo’s Genomic DNA Clean & Concentrator kit (Zymo Research Corporation, Irvine, CA, United States) were used to equally combine and purify the PCR products. Ultimately, using the Illumina HiSeq 2500 platform to sequence the amplicons.

Raw sequences based on 16S rRNA gene were processed with the mothur software package (version 1.30.0, 2013)^2^ according to the MiSeq standard operating procedure (Kozich et al., 2013). To sum up, raw reads were combined, denoised, trimmed, quality-filtered, and aligned to the SILVA 16S version 138 using mothur (Quast et al., 2013). After that, the high-sequences were clustered into ASVs at a rate of 100% similarity level, meanwhile, the lineages belonging to chloroplasts, mitochondria, eukaryotes or unknown were removed. Each of the representative ASV sequences was classified using the SILVA 16S version 138 at the recommended bootstrap threshold of 80% (Wang et al., 2007). All singletons and ASVs occurring in only two samples were excluded from the ASV table in order to minimize bias caused by sequencing depth. Finally, we obtained 4,602 ASVs in ASV table through the entire sample set. Ultimately, the minimum number of sequences in the whole sample was randomly subsampled to correct for differences in sequencing depth, and then the following metrics described below were all based on this final ASV table.

### 2.4 Statistical analyses

The spatial distribution of seawater temperature was visualized by using the Spatial Analyst tool and 3D Analyst tool of ArcToolbox in ArcGIS software. Bacterioplankton community structure was analyzed by performing detrended correspondence analysis (DCA) in the R package of vegan. To determine the existence of regime shift, we drew a linear fitting diagram between DCA1 values and water temperature by using ggplot2 and ggpmisc package in R (Hu et al., 2020). And the critical point estimation of regime shift was according to the linear fitting diagram between DCA1 values and water temperature by using piecewise regression analyses (Hu et al., 2020).

Subsequently, our study had done the analyses to explore the differences among the states of regime shifts. We used the vegan package of R to perform permutational multivariate analysis of variance (PERMANOVE) to test whether the states of regime shift had significant influence on bacterioplankton community structure. Columnar stacking maps about the relative abundance of bacterioplankton were conducted using GraphPad Prism 8 software. The venn plot was performed to verify the shared and unique bacterioplankton ASVs among different states of regime shifts by using the VennDiagram package in R. The redundancy analysis (RDA) was carried out to link bacterioplankton community compositions with environmental variables by using the ggvegan package in R. The relationships of the relative abundance of bacterioplankton phylum, family, and genus with temperature, as well as the relationships of the ASV richness and Shannon index with temperature was assessed by using the ordinary least square (OLS). The heatmaps were performed to show the distributions of the relative abundances of the top 25 genus/families (clades) along temperature gradients using the “pheatmap” package in R. Bacterioplankton community assembly processes were calculated by using iCAMP and treeio packages in R, and visualized by GraphPad Prism8 (Ning et al., 2020).

## 3 Results

### 3.1 The steady-state transformation of bacterioplankton community structure in response to long-term thermal pollution in the Daya Bay

In our study, the winter water temperature in Daya Bay ranged from 19.6°C to 24.12°C, and it was found being the primary environmental factor influencing the compositions of the bacterioplankton community. There were significant positive correlations between temperature and salinity/solubility reactive phosphorus (SRP) and negetive correlations between temperature and pH/NH_4_^+^ (Supplementary Figure S1). Due to long-term thermal pollution from the nuclear power plant, seawater temperature increased in the Daya Bay, leading to a significant temperature difference of 4.5°C between the southern and northern parts of the bay (Figure 1B).

**Figure 2:**
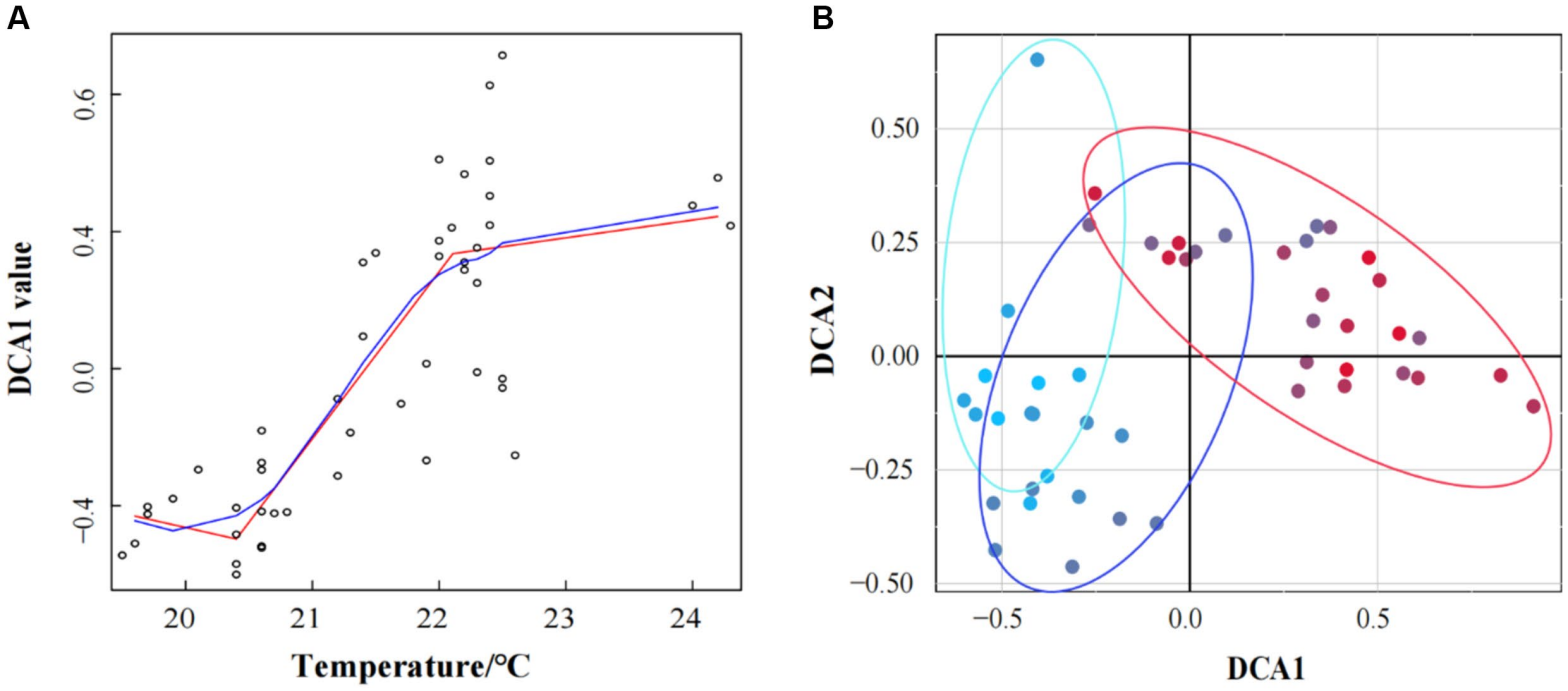
Under temperature conditions, DCA1 values exhibited a regime shift, with critical points of 20.5°C and 22°C. The blue line was a smooth fitting curve between the DCA1 value and water temperature; The red line was a segmented linear regression line that displayed the tipping point **(A)**. Detrended correspondence analysis (DCA) of bacterioplankton community structures in winter of Daya Bay. The blue circle represented samples below 20.5°C, the purple circle represented samples between 20.5°C and 22°C, and the red circle represented samples above 22°C **(B)**.

Linear regression analysis between DCA I axis values and seawater temperature revealed that the steady-state transformation of bacterioplankton community structure in response to seawater temperature increasing aligned with abrupt regime shift of the three typical ecological steady-state transition models (smooth regime shift, abrupt regime shift, and discontinuous regime shift). Critical points were identified at 20.5°C and 22°C, resulting in three stable states: State 1 (<20.5°C), Transition State (TS, 20.5°C < and < 22°C), and State 2 (>22°C) (Figure 2A). In redundancy analysis, the RDA1 axis explained 22.2% of bacterioplankton community structure, and the RDA2 axis explained 9.22%. A significant divergence in bacterioplankton community structure was observed between State 2 and the others, while State 1 and TS also differed on the RDA2 axis (Supplementary Figure S1). Similar findings were observed in detrended correspondence analysis (DCA), showing a significant divergence in the bacterioplankton community compositions according to seawater temperature (Figure 2B).

### 3.2 Alpha diversity patterns of bacterioplankton according to the three states in regime shift

**Figure 3:**
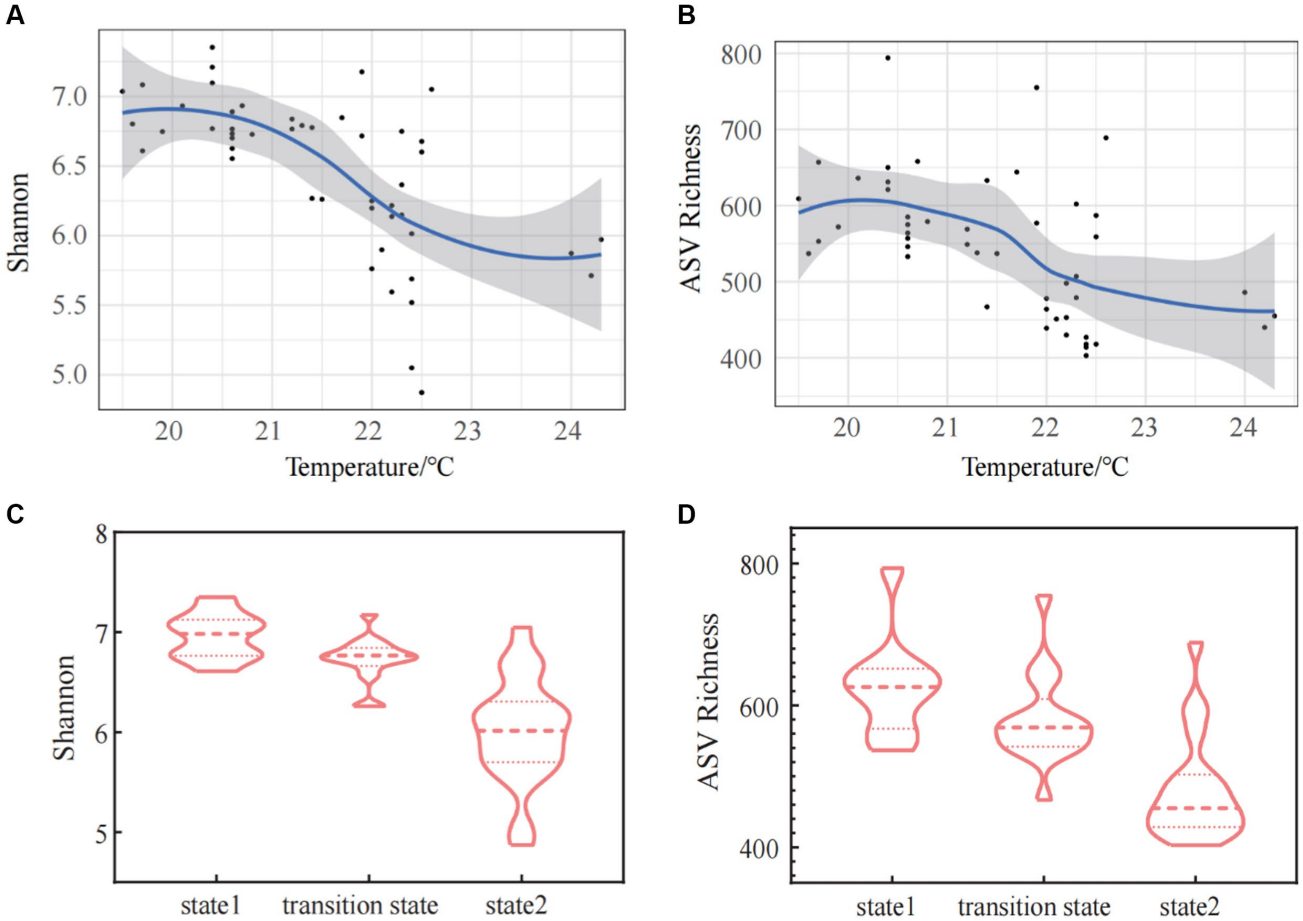
The alpha diversity [Shannon index **(A)** and ASV Richness index **(B)**] of bacterioplankton along the gradient of the water temperature. The changes of Shannon index **(C)** and ASV Richness index **(D)** in three states of the regime shift.

ASV richness index, Shannon index, Simpson index, Pielou index, Chao1 index, and ACE index all exhibited a highly significant negative correlations with temperature (Spearman correlations: *R* = −0.546, *p* < 0.01 for ASV richness index; *R* = −0.674, *p* < 0.01 for Shannon index; *R* = −0.696, *p* < 0.01 for Simpson index; *R* = −0.709, *p* < 0.01 for Pielou index; *R* = −0.552, *p* < 0.01 for Chao1 index; *R* = −0.550, *p* < 0.01 for ACE index) (Table 1). The linear regression curve and the steady-state change curve of alpha diversity indices (Shannon index and ASV Richness index) with seawater temperature fitted well. The alpha diversity of bacterioplankton communities decreased with increasing temperature, accompanied by significant changes during regime shifts (Figures 3A,B). Comparative analyses of alpha diversity indices (Shannon index and ASV Richness) among the three stable states indicated that alpha diversity in state 2 was lower than in TS, and both were lower than in state 1(Figures 3C,D). This suggested a decreasing trend in alpha diversity with increasing seawater temperature.

**Table 1:** The relationships between environmental factors and the alpha diversity of bacterioplankton, including the Spearman correlation statistic’s *R* values and statistical significance, determined through 9,999 permutations.

α diversity	Environment factors	*R* value	*p* value
ASV Richness	T	−0.546	<0.01
Shannon	T	−0.674	<0.01
Simpson	T	−0.696	<0.01
Pielou	T	−0.709	<0.01
Chao1	T	−0.552	<0.01
ACE	T	−0.550	<0.01
Richness	Sal	−0.346	0.016
Shannon	Sal	−0.436	0.002
Simpson	Sal	−0.499	0.0003
Pielou	Sal	−0.472	0.001
Chao1	Sal	−0.349	0.015
ACE	Sal	−0.349	0.015

T, seawater temperature; Sal, Salinity.

### 3.3 The shifts in bacterioplankton community compositions according to the three states of regime shifts

At the phylum level, bacterioplankton phyla with top five highest relative abundance had significant correlations with seawater temperature (Spearman correlations: *R* = 0.570, *p* < 0.01 for Actinobacteriota; *R* = −0.506, *p* < 0.01 for Alphaproteobacteria; *R* = −0.748, *p* < 0.01 for Bacteroidota; *R* = 0.525, *p* < 0.01 for Cyanobacteria; *R* = −0.544, *p* < 0.01 for Gammaproteobacteria) (Supplementary Figure S2; Supplementary Table S1). Among the transition of regime shift, a noticeable change occurred in the compositions of bacterioplankton communities. In state 2, the relative abundance of Gammaproteobacteria, Bacteroidota, Planctomycetota and Alphaproteobacteria was significantly lower than in state 1. In contrast, Cyanobacteria and Actinobacteriota exhibited significantly higher relative abundance in state 2. The transition state exhibited intermediate relative abundance between state 1 and state 2 (Supplementary Figure S3). DCA analysis of the top seven phyla by relative abundance, with linear analysis of DCA I axis data and seawater temperature, showed that the trends of Actinobacteriota, Alphaproteobacteria, Bacteroidota, and Campilobacterota aligned well with abrupt regime shift of the three typical ecological steady-state transition models (Supplementary Figure S4).

**Figure 4:**
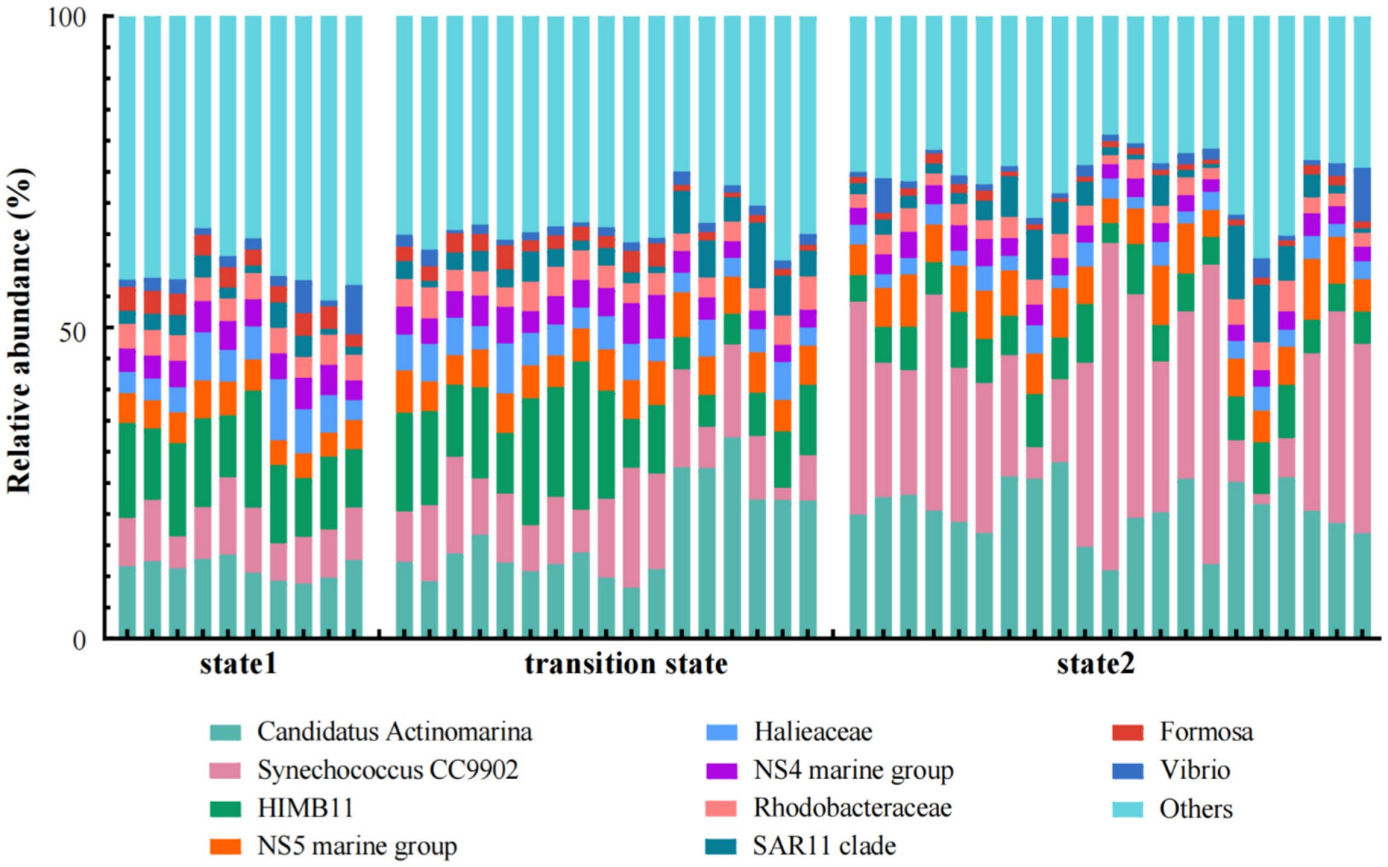
Relative abundances of the dominant bacterial taxa in the three states of regime shift which was separated by water temperature.

**Figure 5:**
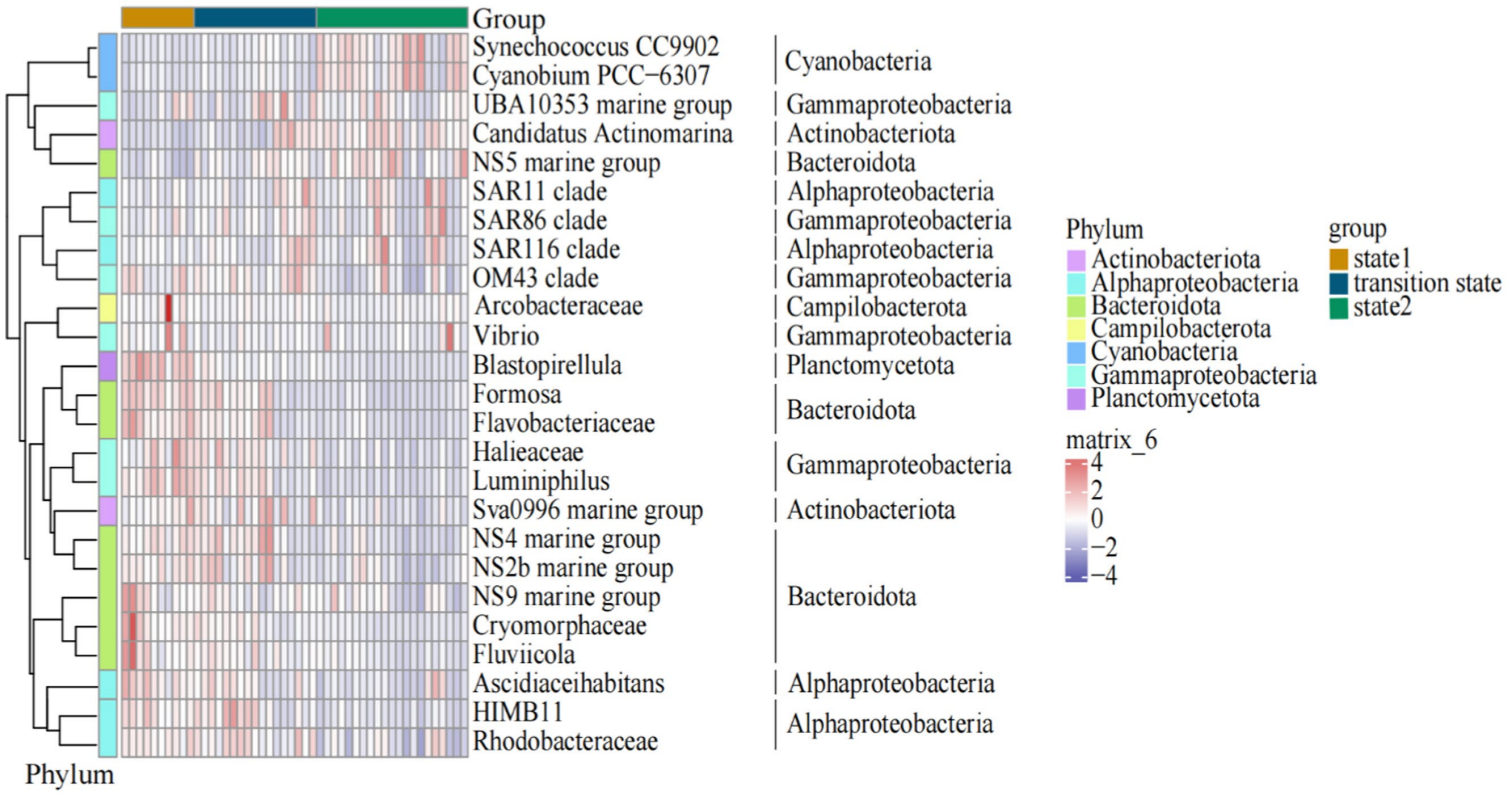
The distributions of the dominant bacterioplankton taxa clades along the gradient of the water temperature in the Daya Bay.

Among the top 10 genus/families (clades) with the highest relative abundance, Formosa and NS4 marine group of Bacteroidota, *Halieaceae* of Gammaproteobacteria, and HIMB11 of Alphaproteobacteria exhibited significant decrease in abundance as the steady state transitioned from state 1 to state 2. In contrast, the relative abundance of NS5 marine group of Bacteroidota, *Synechococcus* of Cyanobacteria, and Candidatus *Actinomarina* of Actinobacteriota increased when the steady state transitioned from state 1 to state 2 (Figure 4). *Arcobacteraceae* of Campilobacterota, *Blastopirellula* of Planctomycetota, *Halieaceae* and Luminiphilus of Gammaproteobacteria, *Ascidiaceihabitans* of Alphaproteobacteria, and NS9 marine group, *Cryomorphaceae*, and *Fluviicola* of Bacteroidota dominated in state 1. In the transition state, the main lineages or clades were mostly composed of the bacteria UBA10353 marine group of Gammaproteobacteria, Sva0996 marine group of Actinobacteriota, NS4 marine group, and NS2b marine group of Bacteroidota, and HIMB11 and *Rhodobacteraceae* of Alphaproteobacteria. Meanwhile, *Synechococcus* and *Cyanobium* of Cyanobacteria, NS5 marine group of Bacteroidota, SAR11 clade and SAR116 clade of Alphaproteobacteria, and SAR86 clade of Gammaproteobacteria dominated in state 2 (Figure 5).

In linear regression analyses, the relative abundance of *Cyanobium* and *Synechococcus* of Cyanobacteria, NS5 marine group of Bacteroidota, and *Vibrio* of Gammaproteobacteria showed a significant positive correlation with seawater temperature. Simultaneously, the relative abundance of *Flavobacteriaceae* of Bacteroidota and *Rhodobacteraceae* of Alphaproteobacteria decreased with increasing seawater temperature. Additionally, SAR11 clade of Alphaproteobacteria, UBA10353 marine group, and SAR86 clade of Gammaproteobacteria showed a lower correlation with seawater temperature. The linear regression curves of these nine genus/families (clades) with temperature were similar, and bacterioplankton relative abundance underwent changes during their regime shifts (Supplementary Figure S6).

Based on the ASV table, a Venn diagram was constructed to show shared and unique ASVs among the three states during regime shifts. The results revealed a total of 934 shared ASVs among the three states, accounting for 20.6% of the total ASVs. State 1 had 791 unique ASVs, representing 17.5%, TS had 1,157 unique ASVs, constituting 25.6%, and state 2 had 1,048 unique ASVs, making up 23.2%. The number of unique ASVs in TS and state 2 was significantly higher than in state 1 (Supplementary Figure S5). Additionally, permutational multivariate analysis of variance showed that BCCs among the three stable states had significant differences (*p* < 0.01), and the differences of the pairwise BCC comparisons among the three stable states were also highly significant (*p* < 0.01) (Table 2).

**Table 2:** Significance tests of bacterioplankton community compositions (BCCs) in three states of regime shift, respectively.

	F model	*R* ^2^	*p* value
State 1 vs. state 2	19.638	0.404	<0.001
State 1 vs. TS	3.830	0.133	0.002
State 2 vs. TS	10.277	0.222	<0.001
Overall	11.328	0.335	0.001

Permutational multivariate analysis of variance (PERMANOVA) based on Bray–Curtis dissimilarity matrices was used. *p* < 0.01. TS, transition state.

### 3.4 The shifts in bacterioplankton community assembly processes according to the three states in regime shift

**Figure 6:**
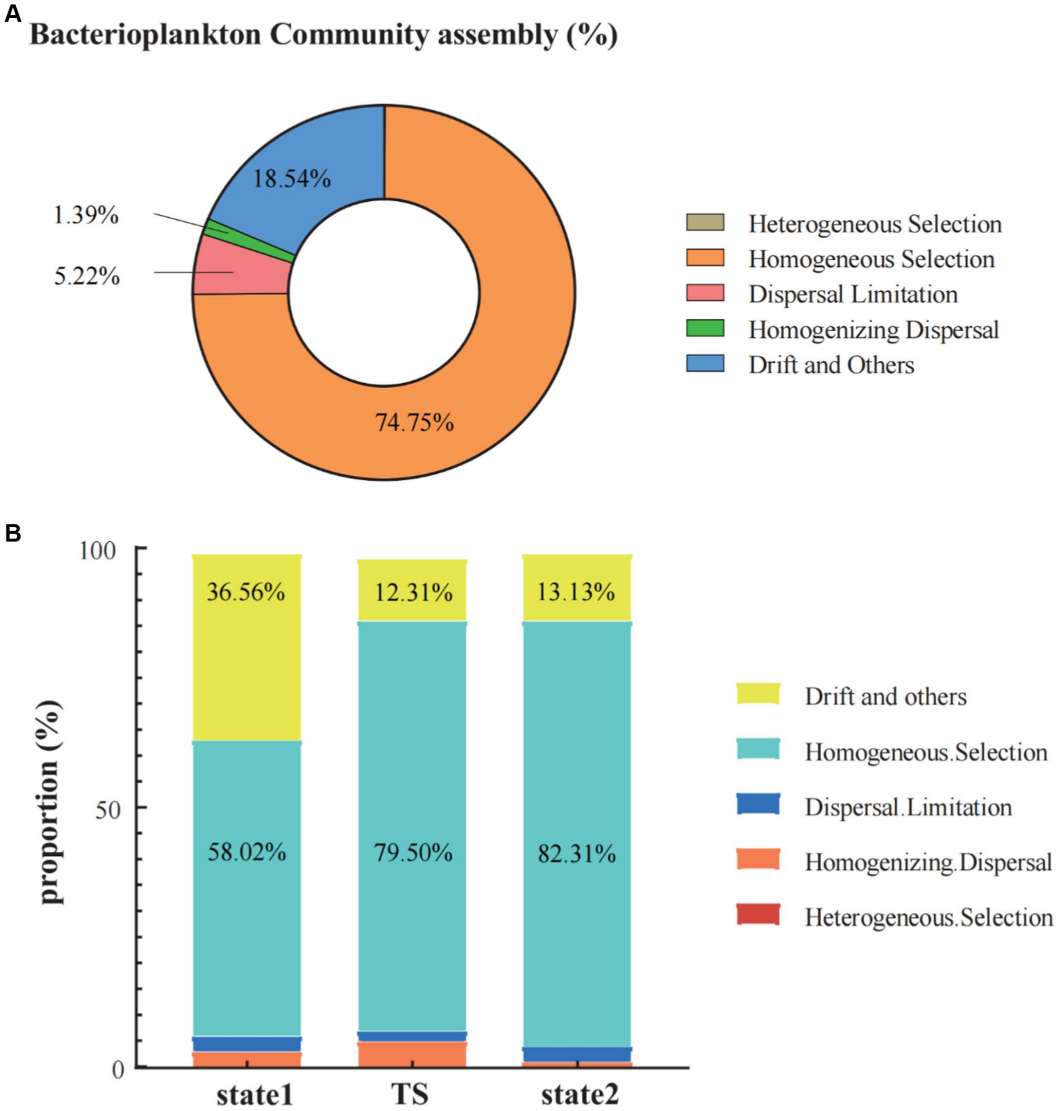
The community assembly processes in the Daya Bay included heterogeneous selection, homogeneous selection, dispersal limitation, homogenizing dispersal and the drift and other fractions **(A)**. The bar chart showed the trend of changes in the proportions of community assembly processes (heterogeneous selection, homogeneous selection, dispersal limitation, homogenizing dispersal and the drift and other) in the three states of the regime shift, respectively **(B)**.

In the three states in regime shift, the processes of the bacterioplankton community assembly (including heterogeneous selection, homogeneous selection, dispersal limitation, homogenizing dispersal, and drift and others) were dominantly characterized by homogeneous selection (74.75%), followed by drift and others (18.54%) and dispersal limitation (5.22%). While, homogenizing dispersal made a smaller contribution (1.39%), and heterogeneous selection almost had no impact (0.10%) (Figure 6A). When calculating the processes of bacterioplankton community assembly by grouping samples based on bacterioplankton regime shift driven by seawater temperature, the results revealed that homogeneous selection consistently dominated in all three states. In the transition of regime shift, the proportion of homogeneous selection increased from 58.02% in state 1 to 79.50% in TS, and reaching to 82.31% in state 2. In contrast, the relative importance of drift and others significantly decreased after the transition of regime shift, dropping from 36.56% in state 1 to below 15% in state 2. Additionally, the proportion of homogenizing dispersal in state 2 was significantly lower than in state 1 and TS (Figure 6B).

## 4 Discussion

The thermal effluents from two nuclear power plants in Daya Bay have been present for more than 20 years and have generated comparatively stable temperature gradients (a temperature increase from 0 to 5°C) in the whole Bay. We found the increase in seawater temperature of the bay as an external driving force caused a steady-state transition of bacterioplankton community structure. This steady-state transition more likely matched an abrupt regime shift rather than a smooth regime or a discontinuous regime model, and it caused significant changes of bacterioplankton community diversity, compositions and structure.

### 4.1 Temperature increasing caused by long-term thermal pollution caused an abrupt regime shift of bacterioplankton community structure in the Daya Bay

Due to Daya Bay being a semi enclosed bay, the long-term thermal effluents from the cooling systems of the Daya Bay Nuclear Power Plant, has significantly impacts on the seawater temperature in Daya Bay, resulting in a substantial temperature difference between the northern and the southern parts of the bay in winter (Figures 1A,B). Our study revealed long-term thermal effluents from nuclear power plants had a strong impact on bacterioplankton community, and beyond a certain temperature critical point, the bacterioplankton community structure underwent a regime shift. Our findings were supported by the existing research, which suggested that water temperature was one of the important environmental factors in shaping aquatic plankton communities (Wang et al., 2010; Berner et al., 2018; Rosero-López et al., 2022). For instance, in the research on the impact of water flow on benthic cyanobacteria, Rosero-López et al. (2022) found that a decrease in water flow can lead to an increase in water temperature, resulting in a significant decrease in benthic cyanobacteria biomass. Upon reaching a certain critical point, the structure and function of benthic communities underwent a sudden shift (Rosero-López et al., 2022).

Our research found that in the winter of the Daya Bay, bacterioplankton community structure underwent a significant transformation when the seawater temperature reached 20.5°C and 22°C, indicating that these temperatures were critical points for the occurrence of regime shifts in bacterioplankton community structure in the Daya Bay (Figure 2A). The study of Berner et al. (2018) on the relationship between seawater temperature and bacterial communities in the Northwest of Gotland Sea (Baltic Proper) showed that changes in temperature directly affect bacterial community composition. An increase in temperature lead to an increase in cyanobacterial biomass and a faster peak (Berner et al., 2018). Therefore, it is confirmed once again that the bacterioplankton community is highly sensitive to changes in water temperature and can produce a rapid response. Moreover, a significant temperature difference was found to easily induce a regime shift in aquatic bacterioplankton community (Wang et al., 2010; Berner et al., 2018; Rosero-López et al., 2022).

### 4.2 The regime shift of bacterioplankton community structure under thermal pollution decreased bacterioplankton alpha diversity

In the aquatic ecosystems, the alpha diversity of the bacterioplankton community (e.g., Shannon index, ASV Richness, etc.) was influenced by various environmental factors. As water temperature could affect the activity of microbial metabolic enzymes directly, the impact of seawater temperature on the alpha diversity of microbial communities was direct and significant (Adams et al., 2010). Especially in coastal areas, changes in water temperature have a filtering effect on microbial communities, influencing their composition and altering their diversity (Trombetta et al., 2019; Lambert et al., 2021; Trombetta et al., 2021). Several studies had confirmed that water temperature can directly or indirectly affect the microbial diversity (Wu et al., 2019; Liu et al., 2023). Research on microorganisms in sediment from the South China Sea indicated that a decrease in temperature can lead to a reduction in bacterial diversity (Wu et al., 2019). The study of Trombetta et al. demonstrated that temperature was the primary driving force for the diversity of bacterioplankton in coastal ecosystems, and the increase in seawater temperature significantly affected the diversity of bacterioplankton communities (Trombetta et al., 2022).

Our research found a significant negative relationship between the alpha diversity of bacterioplankton community (Shannon index, ASV Richness) and the changes in water temperature in the surface seawater of Daya Bay during winter. Many studies had also demonstrated that the microbial richness decreased with increasing temperature (Rao et al., 2021; Grabowska-Grucza et al., 2022; Xing et al., 2022). The correlation analysis between bacterioplankton diversity and temperature showed that there was a significant positive correlation between bacterioplankton diversity and water temperature at 9°C to 19°C, while there was a significant negative correlation between bacterioplankton and water temperature at 19°C to 30°C (Liu et al., 2023). Similarly, in the experiment at geothermal environment, it was also demonstrated that microbial diversity was directly controlled by temperature, and the diversity of microorganisms began to show a decreasing trend at around 20°C (Sharp et al., 2014). This temperature was identified as the critical point for significant changes in the alpha diversity of bacterioplankton communities in aquatic environments. As confirmed by our research, when seawater temperature high than 20.5°C, it caused a regime shift in the alpha diversity of bacterioplankton communities. However, the alpha diversity of bacterioplankton community will not continue to decrease. Instead, it reached a new stable state around 22.5°C (state 2). The diversity change was more apparent in the transition state.

### 4.3 The regime shifts of bacterioplankton community structure under thermal pollution changed bacterioplankton community composition

In our study, specific bacterioplankton taxa were found in each of the three states in the regime shifts (Figures 4, 5; Supplementary Figure S3). One such taxa was *Synechococcus*, supporting previous observations that temperature was an important factor influencing *Synechococcus* distribution (Hamilton et al., 2014; Kolda et al., 2020). Our study showed that the relative abundance of *Synechococcus* in state 2 (> 22°C) was significantly higher than that in state 1 (20.5°C) and TS (from 20.5°C to 22°C) in winter seawater. It was similar to the findings in Kim et al. (2018), which indicated that relative abundance of *Synechococcus* was increased at 24°C compared to an environment temperature of 20°C. When seawater temperature ranging from 21°C to 28°C, the relative abundance of NS5 marine group increased as temperature increasing, showing a significant positive correlation with seawater temperature (Liu et al., 2022). Similarly, in our study, the relative abundance of NS5 marine group was positively correlated with temperature when it in a range of 20°C to 24°C (Supplementary Figure S6). This finding was probably because NS5 marine group preferred environments with high chlorophyll-a concentration and enriched nutrient conditions in the marine ecosystems (Lucas et al., 2015; Díez-Vives et al., 2019; Tong et al., 2021). Our study found that the relative abundance of *Flavobacteriaceae* of Bacteriodota and *Rhodobacteraceae* of Alphaproteobacteria showed significant negative correlations with increasing temperature (Supplementary Figure S6). Similar findings were shown in previous studies that an increase in water temperature can lead to a significant decrease in the richness and the relative abundance of *Flavobacteriaceae* and *Rhodobacteracea*e (Buchan et al., 2014; Gutiérrez et al., 2018). The response of these specific bacterial taxa to the increase in seawater temperature suggested that different bacterioplankton taxa tended to have different optimal temperatures, and led to a regime shift of bacterioplankton community structure under thermal pollution.

### 4.4 The regime shifts of bacterioplankton community structure under thermal pollution changed bacterioplankton community assembly processes

There are several community assembly processes that determined community diversity and dynamics, including heterogeneous selection (HeS), homogeneous selection (HoS), dispersal limitation (DL), homogenizing dispersal (HD), and ‘drift and others’ (DR) (Vellend, 2010; Vellend et al., 2014). In general, homogeneous selection was the main community assembly process (70–80%) of communities in marine surface bacterioplankton community, and stochastic processes had much weaker impact on surface water bodies (Allen et al., 2020). The community assembly process in the surface seawater of Daya Bay was similar to the findings of Allen et al. (2020), which also showed homogeneous selection being the main community assembly process in determining bacterial community assembly. Studies had also shown that moderately eutrophic bays had an inducing effect on the selectivity and stability of bacterioplankton communities, thereby enhancing community structure and deterministic processes, which typically played a more important role than stochastic processes (Dai et al., 2017; Li et al., 2020; Jiao et al., 2021; Wu et al., 2021). Our research drew the same conclusion that, as eutrophic bay, the deterministic process dominated in Daya Bay.

Water temperature was one of the key factors affecting community assembly and the most important environmental modulator for balancing stochastic and deterministic assembly processes (Allen et al., 2020; Zhu et al., 2022; Liu et al., 2023). Our research also confirmed that the impact of changes in water temperature on deterministic and stochastic processes was most evident at the critical point of 20.5°C in regime shift. Studies had confirmed that deterministic processes made a greater contribution to the community assembly of bacterioplankton in warm waters (Chen et al., 2022; Liu et al., 2023). For instance, in previous study, Chen et al. pointed out that deterministic processes dominated the community assembly when the temperature was below 30°C, and stochastic processes dominated the community assembly process when the temperature exceeded 30°C (Chen et al., 2022). In our study, the winter temperature range of seawater in Daya Bay was 19°C to 24°C, and deterministic processes dominated the community assembly of bacterioplankton from low temperature to high temperature environments. Our research also found that when the regime shift from state 1 to TS in the Daya Bay, that was, after the water temperature exceeded 20°C, homogeneous selection increased by 30% in the community assembly, while the proportion of drift and others decreased by about 25%; When the temperature was below 20.5°C, although homogeneous selection accounts for a large proportion, the proportion of drift and others was also high. When the temperature rose to 22°C, there was no significant change in homogeneous selection and ‘drift and others’, but there was a significant decrease in homogenizing dispersal. The strong effect of homogeneous selection is probably because of the extremely high population growth rates of bacteria (Van der Gucht et al., 2007). Bacterioplankton can rapidly track changes in the environmental temperatures (Muylaert et al., 2002). Thus, the persistence of newly arrived bacterioplankton taxa that migrated via flowing water was more likely to be controlled by homogeneous selection than by dispersal limitation or drift. This finding was in line with a previous study conducted on grassland microbial communities in response to experimental warming (Ning et al., 2020). It showed that warming gradually enhanced homogeneous selection which is primarily imposed on Bacillales, but weakened drift in microbial community. In summary, we found high temperatures induced by thermal pollution changed the relative importance of different community assembly processes, and caused a regime shift of bacterioplankton community structure in the subtropical Daya Bay.

## 5 Conclusion

Our study revealed that long-term thermal pollution from the cooling system of the nuclear power plants caused a regime shift of winter bacterioplankton community structure in a subtropical bay. In the three typical scenarios of regime shifts, the steady-state transition of bacterioplankton community structure in response to temperature increasing was more likely consistent with an abrupt regime shift rather than a smooth regime or a discontinuous regime model. The regime shift caused significant changes of bacterioplankton community diversity, compositions and assembly processes. We found bacterioplankton diversity decreased significantly as regime shifts from low-temperature habitats to high-temperature environments. The proportion of homogeneous selection significantly increased, and *Cyanobium*, *Synechococcus*, NS5 marine group and *Vibrio* were found dominating in regime state of high-temperature environments. Our research might broad the understanding of the ecological impact of thermal effluents on subtropical bays.

## Data availability statement

The datasets presented in this study can be found in online repositories. The names of the repository/repositories and accession number(s) can be found below: https://www.ncbi.nlm.nih.gov/, PRJNA961834.

## Author contributions

ZS: Conceptualization, Data curation, Formal analysis, Investigation, Methodology, Resources, Software, Supervision, Validation, Visualization, Writing – original draft, Writing – review & editing. HC: Writing – review & editing. YD: Writing – review & editing. DH: Writing – review & editing. LR: Writing – review & editing, Conceptualization, Data curation, Funding acquisition, Methodology, Project administration, Supervision, Visualization.

## Footnotes

^2^ http://www.mothur.org
